# 729 "Feets" of Strength: How Glabrous Skin Defied the Odds

**DOI:** 10.1093/jbcr/irac012.283

**Published:** 2022-03-23

**Authors:** Megan Gernt, Cassandra O'Rourke

**Affiliations:** Rhode Island Hospital, Attleboro, Massachusetts; Rhode Island Burn Center, Providence, Rhode Island

## Abstract

**Introduction:**

58 year old female with past medical history notable for IDDM with peripheral neuropathy presented to ER after stepping barefoot onto asphalt in August 2020. Patient initially presented to community ER who performed bedside debridement and referred patient to wound clinic for management. After presenting to wound clinic, patient was referred to Burn center. She arrived 10 days after burn and was found to have full thickness burns to the soles of bilateral feet, 3% TBSA. The majority of the soles of both feet were injured. The patient had little residual glabrous skin which was uninjured. Glabrous skin is highly specialized skin and grafting non glabrous skin to the soles of the feet does not have optimal functional outcomes.

**Methods:**

The patient was extremely compliant and willing to do whatever was necessary to maintain function. The patient underwent one debridement, two debridements with allografting, and one debridement with wound vacuum application. Wounds were initially treated with daily silvadene dressings and transitioned to daily damp to dry and finally to daily xeroform dressings. Wounds were measured at each follow up appointment, plan was made to intervene only when wounds stopped showing progress, however this plateau was never reached, and wounds were allowed to heal without any further intervention.

**Results:**

One year post burn, the right sole is fully healed while the left sole remains with small open area. The patient does have significant alterations to her gait pattern. However, the patient has managed to heal her wounds and is functioning independently.

**Conclusions:**

Given patient’s IDDM, her perceived chances of healing a graft was low. Furthermore, her risk of infection and eventual amputation was high. Given a very compliant patient and diligent wound care, the patient was able to achieve significant healing of wounds without the need for amputation. Glabrous skin is specialized skin and grafting non glabrous skin to the soles of the feet does not have optimal functional outcomes. Allowing the patient’s own glabrous skin to pull across the bed and heal has provided for optimal functional outcomes.

